# Electrically modulated attachment and detachment of animal cells cultured on an ITO patterning electrode surface

**DOI:** 10.1186/1753-6561-7-S6-O2

**Published:** 2013-12-04

**Authors:** Sumihiro Koyama

**Affiliations:** 1 Institute of Biogeosciences, Japan Agency for Marine-Earth Science and Technology, 2-15 Natsushima-cho, Yokosuka, 237-0061, Japan

## Background

**Figure 1:**
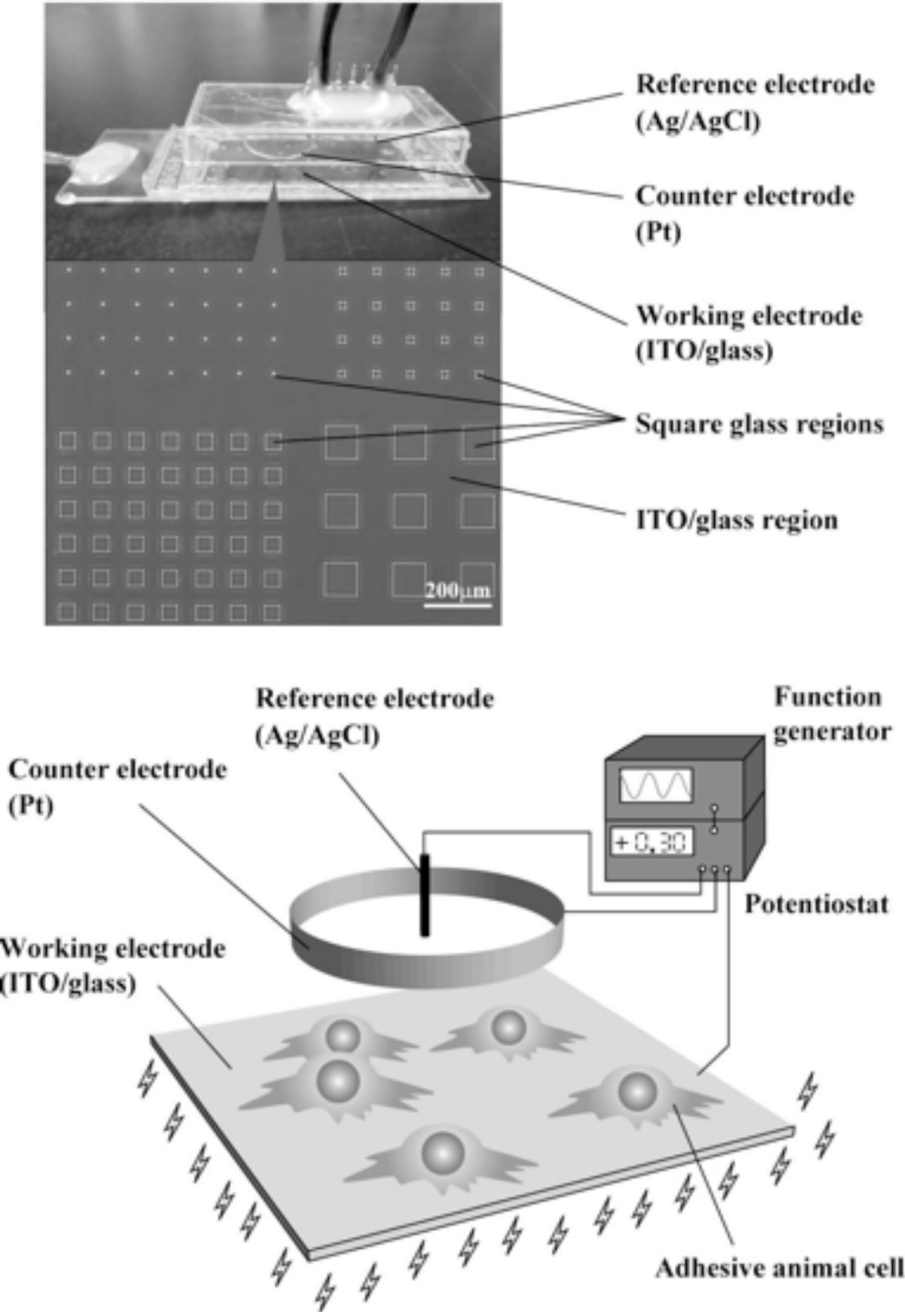
**Schematic illustration of a patterned ITO/glass electrode culture system**.

Micropatterning techniques of animal cells have been reported by numerous groups and fall into 6 major classifications (1). There are 1) photolithography, 2) soft lithography, 3) ink jet printing, 4) electron beam writing, 5) electrochemical desorption of self-assembled monolayers, and 6) dielectrophoresis. These six cell micropatterning techniques cannot modulate both the attachment and detachment of animal cells iteratively at the same positions, however. The present work has demonstrated that a weak electrical potential can modulate the attachment and detachment of specifically positioned adhesive animal cells using a patterned indium tin oxide (ITO)/glass electrode culture system [1], (Figure 1).

## Materials and methods

A patterned indium tin oxide (ITO) optically transparent working electrode was placed on the bottom of a chamber slide with a counter- (Pt) and reference (Ag/AgCl) electrode. The ITO patterning was formed by a reticulate ITO region and arrayed square glass regions of varying size. Constant and rectangular potentials were applied to the working ITO/glass electrode using the Ag/AgCl reference and the Pt counterelectrode (Figure 1). The potentials were delivered via a function generator (AD-8624A, A&D Company, Tokyo, Japan) and a potentiostat (PS-14, Toho Technical Research, Tokyo, Japan).

## Results

Animal cells suspended in serum or sera containing medium were drawn to and attached on a reticulate ITO electrode region to which a +0.4-V vs. Ag/AgCl-positive potential was applied. Meanwhile, the cells were successfully placed on the square glass regions by -0.3-V vs. Ag/AgCl-negative potential application.

Animal cells detached not only from the ITO electrode but also from the square glass regions after the application of a ± 10 mV vs. Ag/AgCl, 9-MHz triangular wave potential in PBS(-) for 30-60 min. The triangular wave potential-induced cell detachment is almost completely noncytotoxic, and no statistical differences between trypsinization and the high frequency wave potential application was observed in HeLa cell growth.

## Conclusions

Using the 3-electrode culture system, the author succeeded in modulation of the attachment and detachment of animal cells on the working electrode surface. The electrical modulation of specifically positioned cell attachment and detachment techniques holds potential for novel optical microscopic cell sorting analysis in lab-on-chip devices.

## References

[B1] Koyama S Electrically modulated attachment and detachment of animal cells cultured on an optically transparent patterning electrode J Biosci Bioeng 2011 7 574 583 (Erratum in: J Biosci Bioeng 2012, **114**: 240-241) 21277827 10.1016/j.jbiosc.2010.12.027

